# Evaluation of the JIA treatment with adalimumab in ukraine

**DOI:** 10.1186/1546-0096-12-S1-P341

**Published:** 2014-09-17

**Authors:** Elena Ponochevna, Elena Okhotnykova

**Affiliations:** 1 Pediatrics, P.L.Shupyk National Medical Academy for Postgraduate Education, Kiev, Ukraine

## Introduction

The prevalence of juvenile idiopathic arthritis (JIA) in Ukrainian children’s population is 4.1 per 10 000 and the total number of children with JIA is about 3000. JIA couldn’t be considered as a benign disease, because very often it results in a sustainable disability. Previously was shown, that early diagnostic and treatment lead to better results. TNF is a key proinflammatory cytokine in the pathogenesis of oligoarthicular and polyarthicular forms of JIA. TNF-inhibition can prevent irreversible structural joint damage. In children with methotrexate failure the after treatment with biologic agents reduces the number of disease flares by 54%. Adalimumab, the anti-TNF-alpha recombinant human monoclonal antibody, recently had been approved in Ukraine for the treatment of polyarthicular juvenil arthritis in children and was included to the state JIA treatment funding programme.

## Objectives

The objective of the study is to summarize the short-term experience of adalimumab treatment in patients with different types of JIA.

## Methods

During the 2013 in the National Children’s Hospital “Ohmatdyt” 79 patients with JIA were treated, aged 5 to 16 years (median -12,5 years), 31 boys and 48 girls. Among girls dominated persistent oligoarthritis and systemic arthritis (50% and 29%, respectively). Boys mostly had polyarthritis, RF-negative and persistent oligoarthritis (48.2% and 22.8%, respectively). 7 children (8.8%) were HLA B27 positive: 6 boys and 1 girl. 7 of all patients were treated with adalimumab and DMARDs. All children on adalimumab were boys with age from 9 to 16 years (adalimumab in Ukraine is approved from 4 y.o.). 6 children had polyarthicular course of JIA, 1 – systemic arthritis. Among them 1 was RF-positive, 1 HLA-B27 positive and 1 had uveitis. RF, HLA-B27 positive patients and patient with systemic arthritis had recurrent courses of disease.

Children with body weight less than 30 kg were given 20mg of adalimumab eow, and if body weight was 30 kg and more – 40mg eow. All patients previously failed to answer to NSAIDs, methotrexate, sulfasalazine or cyclosporine A. The duration from onset of the disease varied from 2 to 10 years. Before the initiation of anti-TNF therapy the TB-infection in all children was excluded by tuberculin and diaskin tests screening.

## Results

In all patients adalimumab has been prescribed in combination with DMARDs as a second line therapy (n=1, time from onset 2 y.) and as a third line therapy (n=6, time from onset 3-10 ys.). On week 16 all patients met ACR Pedi 30 response criteria, mainly due to reduction of acute joint count and physician global assessment of disease activity. In 4 patients the corticosteroid daily dose and in 1 patient the cyclosporine A dosage were reduced. This led to the decrease of hypercorticism’ manifestations and in 2 patients with nanism the tendency to the height’s normalization was outlined. In a child with poliarthritis associated uveitis the stabilization of eye pressure and red blood picture were observed. Two boys still had an active arthritis; one of them was RF+, that is a predictor of poor prognosis. Adalimumab was well tolerated, no skin reactions and infections noticed. As of today the follow up period is 5-8 months and our results are concordant with the data of published randomized clinical trials.

## Conclusion

In patients with JIA adalimumab demonstrated an acceptable safety profile, good tolerance and effectiveness, which led to the reduction of the dosage of potentially harmful concomitant drugs in some cases (glucorticoids and cyclosporine A).

## Disclosure of interest

None declared.

